# Supplementary material for: Facilitating public and patient involvement in basic and preclinical health research
Source: PLoS One. 2019 May 14;14(5):e0216600. doi: 10.1371/journal.pone.0216600 (PMC6516642; doi:10.1371/journal.pone.0216600)
Supplement: S1 File — Supporting methods and tables. (PDF) [file pone.0216600.s001.pdf]

Manuscript title: Facilitating Public and Patient Involvement in basic and preclinical health research.

Authors: James Maccarthy[1], Suzanne Guerin[2], Anthony G. Wilson[1], and Emma R. Dorris[1*]

Affiliations:

1. UCD Centre for Arthritis Research, School of Medicine, UCD Dublin, Ireland
2. UCD School of Psychology, UCD Dublin, Ireland

Corresponding Author: Emma Dorris (emma.dorris@ucd.ie)

Supporting Material

1. Table A: Correlation Matrix from 15 question pilot.
2. Table B: Correlation Matrix following refinement to 8 questions
3. Table C: Pattern Matrix 8Q PAS and GSE
4. Supporting Methods A: Developing a Linked File for Auto-Update of PAS Analysis File
5. Supporting Methods B: Patient Insight Partner Review Guide
6. Supporting Methods C: Pilot Surveys

Table A: Correlation Matrix from 15 question pilot.
Correlations ≥8 are highlighted.

Correlation Matrix[a]

		1)	2)	3)	4)	5)	6)	7)	8)	9)	10)	11)	12)	13)	14)	15)
Correlation	1)	1.000	.798	.570	.490	.530	.364	.492	.511	.459	.356	.521	.423	.495	.461	.581
	2)	.798	1.000	.676	.552	.658	.498	.599	.638	.594	.505	.615	.541	.635	.623	.658
	3)	.570	.676	1.000	.902	.855	.744	.844	.851	.766	.690	.875	.759	.693	.917	.752
	4)	.490	.552	.902	1.000	.893	.773	.798	.800	.804	.678	.842	.779	.681	.869	.691
	5)	.530	.658	.855	.893	1.000	.716	.736	.776	.804	.728	.873	.819	.714	.841	.792
	6)	.364	.498	.744	.773	.716	1.000	.924	.710	.703	.610	.739	.711	.701	.836	.697
	7)	.492	.599	.844	.798	.736	.924	1.000	.812	.730	.625	.794	.728	.689	.836	.757
	8)	.511	.638	.851	.800	.776	.710	.812	1.000	.846	.738	.833	.743	.604	.766	.722
	9)	.459	.594	.766	.804	.804	.703	.730	.846	1.000	.868	.812	.889	.765	.747	.759
	10)	.356	.505	.690	.678	.728	.610	.625	.738	.868	1.000	.727	.797	.652	.639	.629
	11)	.521	.615	.875	.842	.873	.739	.794	.833	.812	.727	1.000	.851	.688	.851	.792
	12)]	.423	.541	.759	.779	.819	.711	.728	.743	.889	.797	.851	1.000	.826	.779	.786
	13)	.495	.635	.693	.681	.714	.701	.689	.604	.765	.652	.688	.826	1.000	.761	.697
	14)	.461	.623	.917	.869	.841	.836	.836	.766	.747	.639	.851	.779	.761	1.000	.759
	15)	.581	.658	.752	.691	.792	.697	.757	.722	.759	.629	.792	.786	.697	.759	1.000
Sig. (1-tailed)	1)		.000	.000	.000	.000	.002	.000	.000	.000	.003	.000	.000	.000	.000	.000
	2)	.000		.000	.000	.000	.000	.000	.000	.000	.000	.000	.000	.000	.000	.000
	3)	.000	.000		.000	.000	.000	.000	.000	.000	.000	.000	.000	.000	.000	.000
	4)	.000	.000	.000		.000	.000	.000	.000	.000	.000	.000	.000	.000	.000	.000

5)	.000	.000	.000	.000		.000	.000	.000	.000	.000	.000	.000	.000	.000	.000
6)	.002	.000	.000	.000	.000		.000	.000	.000	.000	.000	.000	.000	.000	.000
7)	.000	.000	.000	.000	.000	.000		.000	.000	.000	.000	.000	.000	.000	.000
8)	.000	.000	.000	.000	.000	.000	.000		.000	.000	.000	.000	.000	.000	.000
9)	.000	.000	.000	.000	.000	.000	.000	.000		.000	.000	.000	.000	.000	.000
10)	.003	.000	.000	.000	.000	.000	.000	.000	.000		.000	.000	.000	.000	.000
11)	.000	.000	.000	.000	.000	.000	.000	.000	.000	.000		.000	.000	.000	.000
12)	.000	.000	.000	.000	.000	.000	.000	.000	.000	.000	.000		.000	.000	.000
13)	.000	.000	.000	.000	.000	.000	.000	.000	.000	.000	.000	.000		.000	.000
14)	.000	.000	.000	.000	.000	.000	.000	.000	.000	.000	.000	.000	.000		.000
15) [Answer]	.000	.000	.000	.000	.000	.000	.000	.000	.000	.000	.000	.000	.000	.000	

a. Determinant = 8.297E-11

Table B: Correlation Matrix following refinement to 8 questions.

Correlation Matrix[a]

		1)	2)	5)	6)	8)	10)	13)	15)
Correlation	1)	1.000	.798	.530	.364	.511	.356	.495	.581
	2)	.798	1.000	.658	.498	.638	.505	.635	.658
	5)	.530	.658	1.000	.716	.776	.728	.714	.792
	6)	.364	.498	.716	1.000	.710	.610	.701	.697
	8)	.511	.638	.776	.710	1.000	.738	.604	.722
	10)	.356	.505	.728	.610	.738	1.000	.652	.629
	13)	.495	.635	.714	.701	.604	.652	1.000	.697
	15)	.581	.658	.792	.697	.722	.629	.697	1.000

a. Determinant = .001

Table C: Pattern Matrix 8Q PAS and GSE

Pattern Matrix[a]

		Component		
		1	2	3
PAS	1) With the facilities provided? [Answer]	.040	.227	.784
PAS	2) That your needs were considered in the planning of the project meeting(s)? [Answer]	.213	.399	.626
PAS	5) With your level of contribution in relation to what you expected? [Answer]	-.022	.854	.127
PAS	6) With your comfort in voicing your thoughts and opinions? [Answer]	-.100	.905	-.085
PAS	8) With your understanding of the research project and its aims? [Answer]	.079	.862	.007
PAS	10) That you understand the roles of other team members? [Answer]	-.001	.919	-.173
PAS	13) With the communication and feedback tools that you have been asked to use in this project? [Answer]	-.136	.819	.117
PAS	15) That you know who to contact if an issue arises or, if you have concerns about the project or your involvement? [Answer]	.027	.792	.169
GSE	1) I can always manage to solve difficult problems if I try hard enough. [Answer]	.778	.076	-.152
GSE	2) If someone opposes me, I can find the means and ways to get what I want. [Answer]	.389	.122	-.660
GSE	3) It is easy for me to stick to my aims and accomplish my goals. [Answer]	.789	-.044	.085
GSE	4) I am confident that I could deal efficiently with unexpected events. [Answer]	.739	.103	-.285
GSE	5) Thanks to my resourcefulness, I know how to handle unforeseen situations. [Answer]	.861	-.060	-.097

GSE	6) I can solve most problems if I invest the necessary effort. [Answer]	.803	.151	-.070
GSE	7) I can remain calm when facing difficulties because I can rely on my coping abilities. [Answer]	.866	-.050	.063
GSE	8) When I am confronted with a problem, I can usually find several solutions. [Answer]	.827	-.156	.139
GSE	9) If I am in trouble, I can usually think of a solution. [Answer]	.940	-.107	.217
GSE	10) I can usually handle whatever comes my way. [Answer]	.839	.008	-.090

Extraction Method: Principal Component Analysis.

Rotation Method: Promax with Kaiser Normalization.[a]

a. Rotation converged in 5 iterations.

Supporting Methods A: Developing a Linked File for Auto-Update of PAS Analysis File

Create a new blank excel file and change the name to ‘PIP1’.

If you create another linked file, save as ‘PIP2’ etc.

On ‘Sheet1’ create an information table and answer table as follows. Within the answer table, include a GAQ or global assessment question.

	A	B
1	Name	[PIP1 Name]
2	E-mail	[PIP1 E-mail]
3	Phone	[PIP1 Phone]
4		
5	Timepoint	[Insert Date]
6	Question Number	Answer
7	1	5
8	2	5
9	3	7
10	4	7
11	5	7
12	6	5
13	7	5
14	8	10
15	GAQ	7

Figure 1: Information and answer table.

If the research cycle contains multiple time points, create multiple answer tables as below.

	A	B	C	D	E	F	G	H	I	J	K
1	Name	[PIP1 Name]									
2	E-mail	[PIP1 E-mail]									
3	Phone	[PIP1 Phone]									
4											
5	Timepoint	[Insert Date]		Timepoint	[Insert Date]		Timepoint	[Insert Date]		Timepoint	[Insert Date]
6	Question Number	Answer		Question Number	Answer		Question Number	Answer		Question Number	Answer
7	1	5		1	1		1	8		1	5
8	2	5		2	2		2	8		2	6
9	3	7		3	3		3	8		3	5
10	4	7		4	4		4	8		4	6
11	5	7		5	5		5	8		5	5
12	6	5		6	6		6	5		6	4
13	7	5		7	7		7	3		7	5
14	8	10		8	8		8	9		8	4
15	GAQ	7		GAS	9		GAS	7		GAS	6
16											

Figure 2: Multiple answer tables corresponding to multiple time points.

To link 'PIP1' to the 'PAS Analysis Template' file:

In the 'PAS Analysis Template' file, go to the answer table and select the cell corresponding to the answer table in the 'PIP1' file. The value of the content validation question in the 'PAS Analysis Template' file should correspond to the GAQ in the 'PIP1' file.

	Timpoint	[Insert Date]
Question Number	Answer	**FLAGS**
1	=[PIP1.xlsx]Sheet1!B7	Some Attention Required
2	5	Some Attention Required
3	7	No Attention Required
4	7	No Attention Required
5	7	No Attention Required
6	5	Some Attention Required
7	5	Some Attention Required
8	10	No Attention Required
CONTENT VALIDATION	7	PASS

Figure 5: Linking information from 'PIP1' file to a 'PAS Analysis Template' file.

Type '=' and follow the format sequence as displayed in figure 5. Repeat this sequence for question 2-8 and the content validation question.

Alternatively, click and drag the bottom right corner of the green box (as seen in figure 5). This will automatically generate the format sequence for each cell.

Supporting Methods B: Patient Insight Partner Review Guide

Developing a Framework to Assess Public Patient Involvement in Arthritis Research in an Irish Context

Dear Patient Research Partners,

Patients are increasingly sought as research partners and can have active involvement in study design and governance. This is called public and patient involvement (PPI) in research. The aim of this project is to construct a framework to maximise the overall efficiency of public and patient involvement (PPI) in research. To ensure PPI is worthwhile and meaningful for all involved, we want to capture the thoughts and opinions of patient research partners and identify any problems as they arise so that they can be addressed. When I refer to framework, I simply mean the method used to collect these opinions i.e. questionnaire, survey, email or discussion forums. The framework that we are developing is designed for patient research partners involved over the course of a project. We would aim to use this framework ~every 3-6 months during the lifespan of the project, depending on the level of involvement. This framework will also assess the quality of PPI during the course of the research cycle. This is the development stage of the framework. We are sending it to you, our patient research partners, for review and feedback. We will then use your feedback to refine our framework.

Guide for Patient Insight Partners

The framework that we are developing is a questionnaire consisting of a Likert scale. A Likert scale consists of a range of values, in this case, the values range from 1-10. We are not asking you to fill out this framework, we are asking you to review its content, structure and suitability. We ask you to review and critically assess this framework. Your feedback is essential in how the final framework is developed and refined. Please do not hold back in any opinions or criticism as it is extremely valuable. I would like your insight on all aspects. A feedback table consisting of guidelines is provided later in this document.

How to provide your Feedback

A feedback template is provided for you following the framework section at the end of this document. Please fill in this feedback template and remember to save the document to ensure that your feedback is included. The questions are numbered so if you are referencing a particular question in your feedback please identify the question number. Once you have completed your feedback and saved the document, please send the file via email to the following email address james.mac-carthy@ucdconnect.ie by the 16th of July.

Following up on Your Feedback

We would like to discuss this further with you. What is your preferred method of follow up? Please mark your preferred method in the box below.

Group Meeting in UCD	
Individual Meeting in UCD	
External Meeting (geographical limitations apply to this as many	

patient research partners are located throughout the country)	
Phone Call	

Message from the Research Team

On behalf of the research team I would like to thank you for your time and effort. Without your commitment, dedication and hard work this project would not be possible. We hold your input in extremely high regard and I look forward to hearing from you in the coming weeks.

Framework for Review

Question Number	Question								
1	How well do the facilities within the [Research Institute Name] meet your needs? Facilities include parking, physical access, seating etc'. In the box below, please specify any issues that you may have experienced in relation to the facilities within the [Research Institute Name] and suggest how this could be improved.								
1	2	3	4	5	6	7	8	9	10
2	How strongly do you agree/disagree with the following statement? "My needs have been considered in relation to the planning of project meetings, including building access, my availability and facility requirements." 1 indicates you strongly disagree and 10 indicates that you strongly agree. If your needs were not considered, please elaborate in the box below and suggest how this issue could be improved.								
1	2	3	4	5	6	7	8	9	10

<table>
<tr><td colspan="2">3</td><td colspan="8">How easy was the [Research Institute Name] to find? This includes the commute to the UCD campus and finding the Conway Institute once you arrived on the campus. In the box below, please specify any issues you may have had in finding the Conway Institute and suggest how this could be improved.</td></tr>
<tr><td>1</td><td>2</td><td>3</td><td>4</td><td>5</td><td>6</td><td>7</td><td>8</td><td>9</td><td>10</td></tr>
<tr><td colspan="10">This question will only be included in the first questionnaire and will not feature in the following questionnaires.</td></tr>
</table>

<table>
<tr><td colspan="2">4</td><td colspan="8">How strongly do you agree/disagree with the following statement? ”I feel as though my input into the research cycle is valued by the research team and I feel valued as a person.” 1 indicates you strongly disagree and 10 indicates that you strongly agree. If you do not feel valued by the research team, please specify why in the box below and suggest how this could be improved.</td></tr>
<tr><td>1</td><td>2</td><td>3</td><td>4</td><td>5</td><td>6</td><td>7</td><td>8</td><td>9</td><td>10</td></tr>
<tr><td colspan="10"></td></tr>
</table>

<table>
<tr><td colspan="2">5</td><td colspan="8">How strongly do you agree/disagree with the following statement? “I remain committed and motivated to contribute to the research cycle to the best of my ability.” 1 indicates you strongly disagree and 10 indicates that you strongly agree. If you do not feel committed or motivated please specify why in the box below and suggest how this could be improved.</td></tr>
<tr><td>1</td><td>2</td><td>3</td><td>4</td><td>5</td><td>6</td><td>7</td><td>8</td><td>9</td><td>10</td></tr>
<tr><td colspan="10"></td></tr>
</table>

6	How strongly do you agree/disagree with the following statement? “My level of contribution is as expected based on the patient research partner terms of reference.” 1 indicates you strongly disagree and 10 indicates that you strongly agree. If your level of contribution has not been as you expected, please elaborate in the box below and suggest how this issue could be improved.								
1	2	3	4	5	6	7	8	9	10

7	How comfortable do you feel voicing your thoughts and opinions to researchers and other stakeholders involved in the project? 1 indicates very uncomfortable, 10 indicates very comfortable. If you are uncomfortable, please elaborate in the box below and suggest how this issue could be improved.								
1	2	3	4	5	6	7	8	9	10

8	How strongly do you agree/disagree with the following statement? “Members of the the research team are approachable and I am comfortable asking the team about any aspects of the project that I do not understand.” 1 indicates that you strongly disagree, 10 indicates that you strongly agree. If you are uncomfortable about approaching the research team, please specify why in the box below and suggest how this issue could be improved.								
1	2	3	4	5	6	7	8	9	10

<table>
<tr><td>9</td><td colspan="9">How well do you understand the research project and its aims? 1 indicates that you have a very poor understanding, 10 indicates that you have a very clear understanding. If you do not understand the research cycle, its aims or any other aspect of the research cycle, please elaborate in the box below and suggest how this issue could be improved.</td></tr>
<tr><td>1</td><td>2</td><td>3</td><td>4</td><td>5</td><td>6</td><td>7</td><td>8</td><td>9</td><td>10</td></tr>
<tr><td colspan="10"></td></tr>
<tr><td>10</td><td colspan="9">How strongly do you agree/disagree with the following statement? “I clearly understand my specific role within this research cycle.” 1 indicates that you strongly disagree, 10 indicates that you strongly agree. If you do not understand your role, or if any aspects of your role in the project are unclear, please elaborate in the box below and suggest how this issue could be improved.</td></tr>
<tr><td>1</td><td>2</td><td>3</td><td>4</td><td>5</td><td>6</td><td>7</td><td>8</td><td>9</td><td>10</td></tr>
<tr><td colspan="10"></td></tr>
<tr><td>11</td><td colspan="9">How well do you understand the roles of other project partners? If you do not understand the roles of other project partners, please elaborate in the box below and suggest how this issue could be improved.</td></tr>
<tr><td>1</td><td>2</td><td>3</td><td>4</td><td>5</td><td>6</td><td>7</td><td>8</td><td>9</td><td>10</td></tr>
<tr><td colspan="10"></td></tr>
</table>

12	How strongly do you agree/disagree with the following statement? "The research team have provided me with clear and transparent communication in regards to the research project." 1 indicates that you strongly disagree, 10 indicates that you strongly agree. If you fell that the communication of the research team has not been clear and transparent, please elaborate in the box below and suggest how this issue could be improved.								
1	2	3	4	5	6	7	8	9	10

13	How clear are the instructions provided to you? If instructions are not clearly provided, please suggest a way to improve this issue.								
1	2	3	4	5	6	7	8	9	10

14	How strongly do you agree/disagree with the following statement? "The communication and feedback tools that I have been asked to use in this project have been easy to use". 1 indicates that you strongly disagree, 10 indicates that you strongly agree. If you have had any issues regarding the use of communication and feedback tools, please elaborate in the box below and suggest how this issue could be improved.								
1	2	3	4	5	6	7	8	9	10

15	"I have been treated fairly by the research team and by other patient research partners during the research cycle." How strongly do you agree/disagree with this statement? 10 suggests you strongly agree, 1 suggests you strongly disagree. If you feel that you have not been treated fairly, please specify why in the box below and suggest how this could be improved.								
1	2	3	4	5	6	7	8	9	10

16	How strongly do you agree/disagree with the following statement? "Adequate training has been provided to me to facilitate my full participation in this project." 1 indicates that you strongly disagree, 10 indicates that you strongly agree. If you feel that adequate training has not been provided, please elaborate in the box below and suggest how this issue could be improved.								
1	2	3	4	5	6	7	8	9	10

17	How strongly do you agree/disagree with the following statement? "I know who to contact if an issue arises or if I have concerns about the project or my involvement". 1 indicates that you strongly disagree, 10 indicates that you strongly agree. If you are unclear about who to contact should an issue arise, please elaborate in the box below and suggest how this issue could be improved.								
1	2	3	4	5	6	7	8	9	10

18	How strongly do you agree/disagree with the following statement? "When an issue or concern arises, the project team are effective and successful in addressing it". 1 indicates that you strongly disagree, 10 indicates that you strongly agree. If you feel that the research team has not been effective or successful in dealing with issues, please elaborate in the box below and suggest how this issue could be improved.								
1	2	3	4	5	6	7	8	9	10

19	Overall, how satisfied do you feel with your participation in the research cycle? 1 indicates that you very unsatisfied, 10 indicates that you are very satisfied. If you are not satisfied with your participation, please elaborate in the box below and suggest how this issue could be improved.								
1	2	3	4	5	6	7	8	9	10

Feedback Section

Please find the feedback section below. The feedback box is only a template, please feel free to provide feedback on any aspects you like. Your feedback can be as long or as short as you like although we would encourage you to go into as much detail as possible. Your feedback can be in any style you like i.e. bullet points or essay style.

**Feedback Aspect**	**Feedback**
Do you understand the language used?	
Can any questions be phrased better?	
We want to reduce this long list of questions to a shorter list.<br>Which questions are the most relevant?	
Which questions are the least relevant?	
Are any of the questions	

unnecessary?	
Have any important aspects been left out? If so what are they?	
Overall thoughts and opinions?	
If you were a patient research partner, would you be likely to complete the questionnaire every 3-6 months? If no, what can we do to improve the likelihood of completing it?	
Is a 1-10 range appropriate? would a 1-5 or 1-100 be better?	
Is the questionnaire too long, too short?	
Do you think this framework is useful and necessary? Will it capture the information necessary to improve the efficiency and meaningfulness of patient involvement throughout the lifespan of a given research project?	
Is a questionnaire the best way to evaluate your opinion of involvement in a research project? Keep in mind that this is designed to be used throughout a project and not just at the start & end. The goal is to adjust and progress the satisfaction and efficiency of PPI as it happens.	
Can you suggest an alternative method to evaluate the views and opinions of patient research partners?	
Feedback on any other aspects.	

REMINDER: Once you have completed your feedback, save this document to ensure that your feedback is not lost. We would also kindly remind you to mark what your preferred method of follow up is in the table on the second page.

Patient or Public Insight Partner Survey

Have you attended a meeting or event(s) that gave you the opportunity to discuss or express your views about health research or to share your experience with researchers? If so, we need your help to improve patient/public involvement in research.

We are dedicated to making sure that patient and public involvement in research is as successful and meaningful as possible for all involved. We therefore want to develop and use standard ways to assess public involvement in research. We have developed a questionnaire, in association with patient research partners, to help us do this. In order to test how valid our questionnaire is, we ask you to fill in two questionnaires.

Both questionnaires are contained within this survey. This should take about 10 minutes in total.

On behalf of the research team I would like to thank you for your time and effort. We hold your input in extremely high regard and without your commitment, dedication and hard work this project would not be possible.

If you require any further information, please contact the project lead Dr. Emma Dorris (emma.dorris@ucd.ie).

This project is funded by the Health Research Board (HRB).

*Required

Consent

Answers to this section is required.

Please note this survey is completely voluntary and you may withdraw at any time. We do not collect any other information except that which you enter on this form.

1. **Are you over 18?** *
   Mark only one oval.
   - ( ) Yes
   - ( ) No *After the last question in this section, stop filling in this form.*

2. **Do you consent to the non-commercial use of the information you provide?** *
   Mark only one oval.
   - ( ) Yes
   - ( ) No *After the last question in this section, stop filling in this form.*

3. **Have you attended a meeting or event(s) that gave you the opportunity to discuss or express your views about health research or to share your experience with researchers?** *
   Mark only one oval.
   - ( ) Yes
   - ( ) No *After the last question in this section, stop filling in this form.*

4. **If you selected "Yes" to the above question, did you attend that meeting/event as a:** *
   Mark only one oval.
   - ( ) Patient
   - ( ) Carer
   - ( ) Family Member
   - ( ) Interested Public
   - ( ) Researcher

Survey 1

We use the term "project" to refer to any research event you attended in which you had an opportunity to interact with researchers. Please answer the following questions based on your experience at these project(s)/event(s).

In response to the following questions, please indicate your level of satisfaction. 0 = very dissatisfied, 5 = neither satisfied nor dissatisfied, 10 = very satisfied.

How satisfied are you:

5. **1) With the facilities provided?** *
Facilities include parking, physical access, seating etc.
Mark only one oval per row.

	0 - Very Dissatisfied	1	2	3	4	5 - Neither dissatisfied nor satisfied	6	7	8	9	10 - Very Satisfied
Answer	◯	◯	◯	◯	◯	◯	◯	◯	◯	◯	◯

6. **2) That your needs were considered in the planning of the project meeting(s)?** *
Considerations may include building access, your availability and facility requirements.
Mark only one oval per row.

	0 - Very Dissatisfied	1	2	3	4	5 - Neither Dissatisfied nor Satisfied	6	7	8	9	10 - Very Satisfied
Answer	◯	◯	◯	◯	◯	◯	◯	◯	◯	◯	◯

7. **3) That the project team value you and your input?** *
Mark only one oval per row.

	0 - Very Dissatisfied	1	2	3	4	5 - Neither Dissatisfied nor Satisfied	6	7	8	9	10 - Very Satisfied
Answer	◯	◯	◯	◯	◯	◯	◯	◯	◯	◯	◯

8. **4) That you remain motivated to contribute to the research project?** *
Mark only one oval per row.

	0 - Very Dissatisfied	1	2	3	4	5 - Neither Dissatisfied nor Satisfied	6	7	8	9	10 - Very Satisfied
Answer	◯	◯	◯	◯	◯	◯	◯	◯	◯	◯	◯

9. **5) With your level of contribution in relation to what you expected?** *
Mark only one oval per row.

	0 - Very Dissatisfied	1	2	3	4	5 - Neither Dissatisfied nor Satisfied	6	7	8	9	10 - Very Satisfied
Answer	◯	◯	◯	◯	◯	◯	◯	◯	◯	◯	◯

10. **6) With your comfort in voicing your thoughts and opinions?** *
Mark only one oval per row.

	0 - Very Dissatisfied	1	2	3	4	5 - Neither Dissatisfied nor Satisfied	6	7	8	9	10 - Very Satisfied
Answer	◯	◯	◯	◯	◯	◯	◯	◯	◯	◯	◯

11. **7) With your comfort in approaching members of the project team or asking about aspects of the project that you do not understand?** *
Mark only one oval per row.

	0 - Very Dissatisfied	1	2	3	4	5 - Neither Dissatisfied nor Satisfied	6	7	8	9	10 - Very Satisfied
Answer	◯	◯	◯	◯	◯	◯	◯	◯	◯	◯	◯

12. **8) With your understanding of the research project and its aims?** *
Mark only one oval per row.

	0 - Very Dissatisfied	1	2	3	4	5 - Neither Dissatisfied nor Satisfied	6	7	8	9	10 - Very Satisfied
Answer	◯	◯	◯	◯	◯	◯	◯	◯	◯	◯	◯

13. **9) That you understand your specific role within the project team?** *
Mark only one oval per row.

	0 - Very Dissatisfied	1	2	3	4	5 - Neither Dissatisfied nor Satisfied	6	7	8	9	10 - Very Satisfied
Answer											

14. **10) That you understand the roles of other team members?** *
Mark only one oval per row.

	0 - Very Dissatisfied	1	2	3	4	5 - Neither Dissatisfied nor Satisfied	6	7	8	9	10 - Very Satisfied
Answer											

15. **11) That you have received clear and transparent communication from the project team?** *
Mark only one oval per row.

	0 - Very Dissatisfied	1	2	3	4	5 - Neither Dissatisfied nor Satisfied	6	7	8	9	10 - Very Satisfied
Answer											

16. **12) That you have been provided with clear information to carry out your role?** *
Mark only one oval per row.

	0 - Very Dissatisfied	1	2	3	4	5 - Neither Dissatisfied nor Satisfied	6	7	8	9	10 - Very Satisfied
Answer											

17. **13) With the communication and feedback tools that you have been asked to use in this project?** *
Mark only one oval per row.

	0 - Very Dissatisfied	1	2	3	4	5 - Neither Dissatisfied nor Satisfied	6	7	8	9	10 - Very Satisfied
Answer											

18. **14) That you have been treated fairly during the research project?** *
Mark only one oval per row.

	0 - Very Dissatisfied	1	2	3	4	5 - Neither Dissatisfied nor Satisfied	6	7	8	9	10 - Very Satisfied
Answer											

19. **15) That you know who to contact if an issue arises or, if you have concerns about the project or your involvement?** *
Mark only one oval per row.

	0 - Very Dissatisfied	1	2	3	4	5 - Neither Dissatisfied nor Satisfied	6	7	8	9	10 - Very Satisfied
Answer											

20. **16) Overall, how satisfied/dissatisfied are you with your involvement in this project?** *
Mark only one oval per row.

	0 - Very Dissatisfied	1	2	3	4	5 - Neither Dissatisfied nor Satisfied	6	7	8	9	10 - Very Satisfied
Answer											

Survey 2

This survey is not related to a specific project or research event. Please select the answer that best describes you in your day-to-day approach to tasks.

In response to the following statements, please select the answer which is most true to you.

21. **1) I can always manage to solve difficult problems if I try hard enough.** *
Mark only one oval per row.

	1 - Not at all true	2 - Hardly true	3 - Moderately true	4 - Exactly true
Answer	◯	◯	◯	◯

22. **2) If someone opposes me, I can find the means and ways to get what I want.** *
Mark only one oval per row.

	1 - Not at all true	2 - Hardly true	3 - Moderately true	4 - Exactly true
Answer	◯	◯	◯	◯

23. **3) It is easy for me to stick to my aims and accomplish my goals.** *
Mark only one oval per row.

	1 - Not at all true	2 - Hardly true	3 - Moderately true	4 - Exactly true
Answer	◯	◯	◯	◯

24. **4) I am confident that I could deal efficiently with unexpected events.** *
Mark only one oval per row.

	1 - Not at all true	2 - Hardly true	3 - Moderately true	4 - Exactly true
Answer	◯	◯	◯	◯

25. **5) Thanks to my resourcefulness, I know how to handle unforeseen situations.** *
Mark only one oval per row.

	1 - Not at all true	2 - Hardly true	3 - Moderately true	4 - Exactly true
Answer	◯	◯	◯	◯

26. **6) I can solve most problems if I invest the necessary effort.** *
Mark only one oval per row.

	1 - Not at all true	2 - Hardly true	3 - Moderately true	4 - Exactly true
Answer	◯	◯	◯	◯

27. **7) I can remain calm when facing difficulties because I can rely on my coping abilities.** *
Mark only one oval per row.

	1 - Not at all true	2 - Hardly true	3 - Moderately true	4 - Exactly true
Answer	◯	◯	◯	◯

28. **8) When I am confronted with a problem, I can usually find several solutions.** *
Mark only one oval per row.

	1 - Not at all true	2 - Hardly true	3 - Moderately true	4 - Exactly true
Answer	◯	◯	◯	◯

29. **9) If I am in trouble, I can usually think of a solution.** *
Mark only one oval per row.

	1 - Not at all true	2 - Hardly true	3 - Moderately true	4 - Exactly true
Answer	◯	◯	◯	◯

30. **10) I can usually handle whatever comes my way.** *
Mark only one oval per row.

	1 - Not at all true	2 - Hardly true	3 - Moderately true	4 - Exactly true
Answer	◯	◯	◯	◯

Powered by
Google Forms
